# Spontaneous gastrocutaneous fistula presenting as a long-standing abdominal wall nodule: a case report

**DOI:** 10.3389/fmed.2026.1860673

**Published:** 2026-06-04

**Authors:** Qiu-Shi Huang, Ran Huang, Gang Xiao, Jian Shen, Shuo-Yang Huang, Shan He, Jun Bu, Xian-Zhe Yu

**Affiliations:** 1 Department of Gastrointestinal Surgery, West China School of Medicine, Sichuan University, Sichuan University Affiliated Chengdu Second People’s Hospital, Chengdu Second People’s Hospital, Chengdu, China; 2 Department of Pathology, West China School of Medicine, Sichuan University, Sichuan University Affiliated Chengdu Second People’s Hospital, Chengdu Second People’s Hospital, Chengdu, China; 3 Department of Cardiovascular Surgery, West China Hospital, Sichuan University, Chengdu, China

**Keywords:** benign gastric ulcer, case report, enterocutaneous fistula, penetrating ulcers, spontaneous gastrocutaneous fistula, surgical resection

## Abstract

**Background:**

Spontaneous gastrocutaneous fistula (SGCF) is an extremely uncommon clinical condition. In modern practice, most gastro-abdominal fistulas are acquired, typically arising from iatrogenic causes, major abdominal trauma, or direct invasion by advanced gastrointestinal malignancies. SGCF resulting exclusively from benign chronic gastric ulcer penetration is rarely reported and is generally regarded as a historical curiosity.

**Case presentations:**

An 81-year-old man presented with persistent enteric-like drainage after traumatic disruption of a subcutaneous abdominal nodule that had been stable for approximately three decades. Comprehensive imaging and endoscopic investigations confirmed the presence of an SGCF originating from a benign gastric ulcer. Laparoscopic exploration demonstrated dense, localized fibroinflammatory adhesions anchoring the stomach to the anterior abdominal wall. Definitive management consisted of *en bloc* excision of the fistulous tract combined with a laparoscopically assisted subtotal gastrectomy, which was completed successfully.

**Conclusion:**

Although rare, benign penetrating gastric ulcers may give rise to chronic fistulous tracts involving the abdominal wall. In the present case, the imaging, operative, and histopathological findings supported a chronic localized fistulizing process in which the formation of dense adhesions may have limited free perforation. After careful exclusion of malignancy, mature fibrotic fistulas of this type generally require definitive en bloc surgical resection.

## 1 Introduction

Peptic ulcer disease most commonly manifests through well-recognized acute gastrointestinal complications, such as perforation, bleeding, and obstruction of the gastric outlet (1). Posteriorly penetrating ulcers frequently extend into adjacent solid organs such as the pancreas or liver, whereas anteriorly located ulcers typically lead to free perforation with diffuse peritonitis, a life-threatening emergency (2). In contrast, spontaneous gastrocutaneous fistula (SGCF), defined as a direct and mature tract connecting the gastric lumen to the external skin surface, often lined by epithelium or granulation tissue, is exceedingly rare (3).

The increased application of proton pump inhibitors and treatments for eradicating *Helicobacter pylori* (4) has led to a marked decline in the incidence of ulcer-related complications. Against this background, gastrocutaneous fistulas encountered in current clinical settings are almost always acquired (5). They most frequently occur as complications following removal of percutaneous endoscopic gastrostomy tubes, postoperative leaks after bariatric procedures (6), severe abdominal trauma (7), or direct invasion by advanced malignancies such as gastric carcinoma (8). As such, SGCF arising solely from a benign gastric ulcer is highly unusual. In this report, we describe an exceptional case of SGCF caused by a benign gastric ulcer, distinguished by an indolent 30-year history of an abdominal wall nodule that eventually ruptured. We also discuss the underlying pathophysiology and relevant management considerations based on existing literature.

## 2 Case presentations

### 2.1 Patient information and clinical findings

**FIGURE 1:**
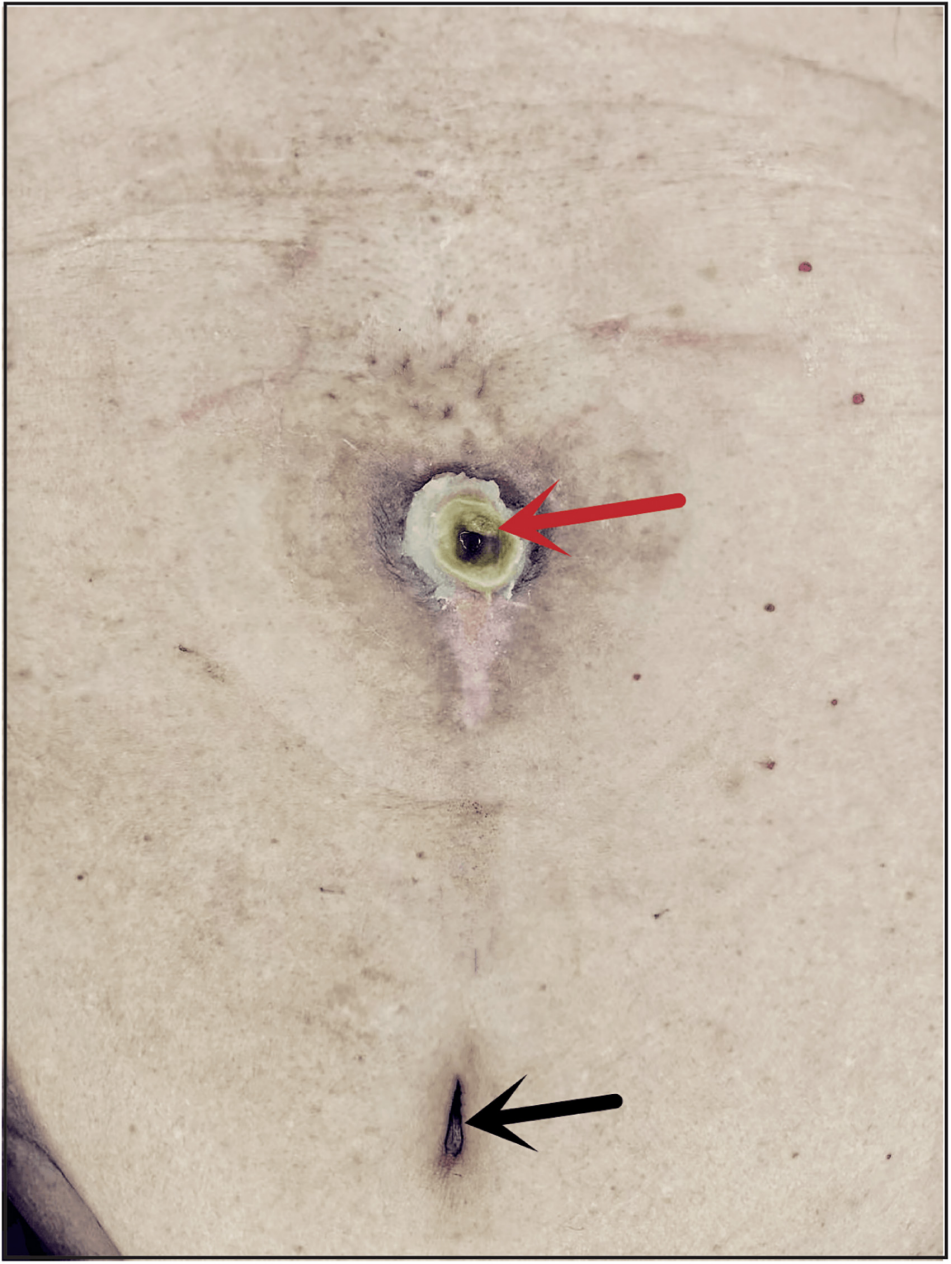
Clinical presentation of the abdomen. The red arrow indicates the external cutaneous orifice (∼0.5 cm) of the fistula with mild erythema. The black arrow points to the umbilicus.

The patient was an 81-year-old man of Han Chinese descent with a 1-month history of persistent fluid discharge from a nodule located in the mid-upper abdomen. His family history was unremarkable, with no known first-degree relatives suffering from gastrointestinal malignancies, nor any documented familial history of *H. pylori* infection. Notably, he was a lifelong non-smoker and had no history of alcohol consumption. He had first noticed a small, painless, and well-defined subcutaneous mass in the epigastric region approximately 30 years earlier. Because the lesion remained stable and asymptomatic over time, he did not seek medical evaluation. Consequently, there was no clinical documentation or photographic evidence of the nodule prior to its external fistulation available for comparison. One month prior to presentation, the lesion was inadvertently traumatized, leading to local skin breakdown, erythema, and swelling, followed by continuous drainage. The discharged fluid was turbid and pale yellow, containing visible food debris, with an estimated daily output of up to 500 mL. The patient denied fever, significant abdominal pain, nausea, melena, or unintended weight loss. His medical history was notable only for an appendectomy performed 40 years earlier. This prior surgery was an open appendectomy via a right lower quadrant McBurney incision for acute appendicitis. The postoperative recovery was uncomplicated, suggesting that it was unlikely to be the primary cause of the dense upper abdominal adhesions observed during the current presentation. The patient denied any past or present use of non-steroidal anti-inflammatory drugs (NSAIDs). Furthermore, he had not experienced symptoms associated with peptic ulcer disease, such as epigastric pain, dyspepsia, or gastrointestinal bleeding, when the nodule had first appeared 30 years before. On examination, despite the substantial ongoing loss of fluid over the preceding month, his hemodynamic status remained fully stable (blood pressure 125/75 mmHg, heart rate 78 beats/min). His body mass index (BMI) was 29.4 kg/m^2^, and his serum albumin level was 35 g/L, suggesting relative preservation of his nutritional status. Laboratory blood tests revealed a hemoglobin level of 14.5 g/dL, and minor electrolyte imbalances that were corrected with intravenous fluid administration; no significant increases in the levels of inflammatory markers were observed. A small cutaneous opening, approximately 0.5 cm in diameter, was observed in the mid-upper abdomen, surrounded by mild erythema. A stoma appliance placed over the lesion collected enteric-appearing fluid. The abdomen was soft, non-distended, and non-tender, with no rebound tenderness or palpable masses (Figure 1).

### 2.2 Diagnostic assessment

**FIGURE 2:**
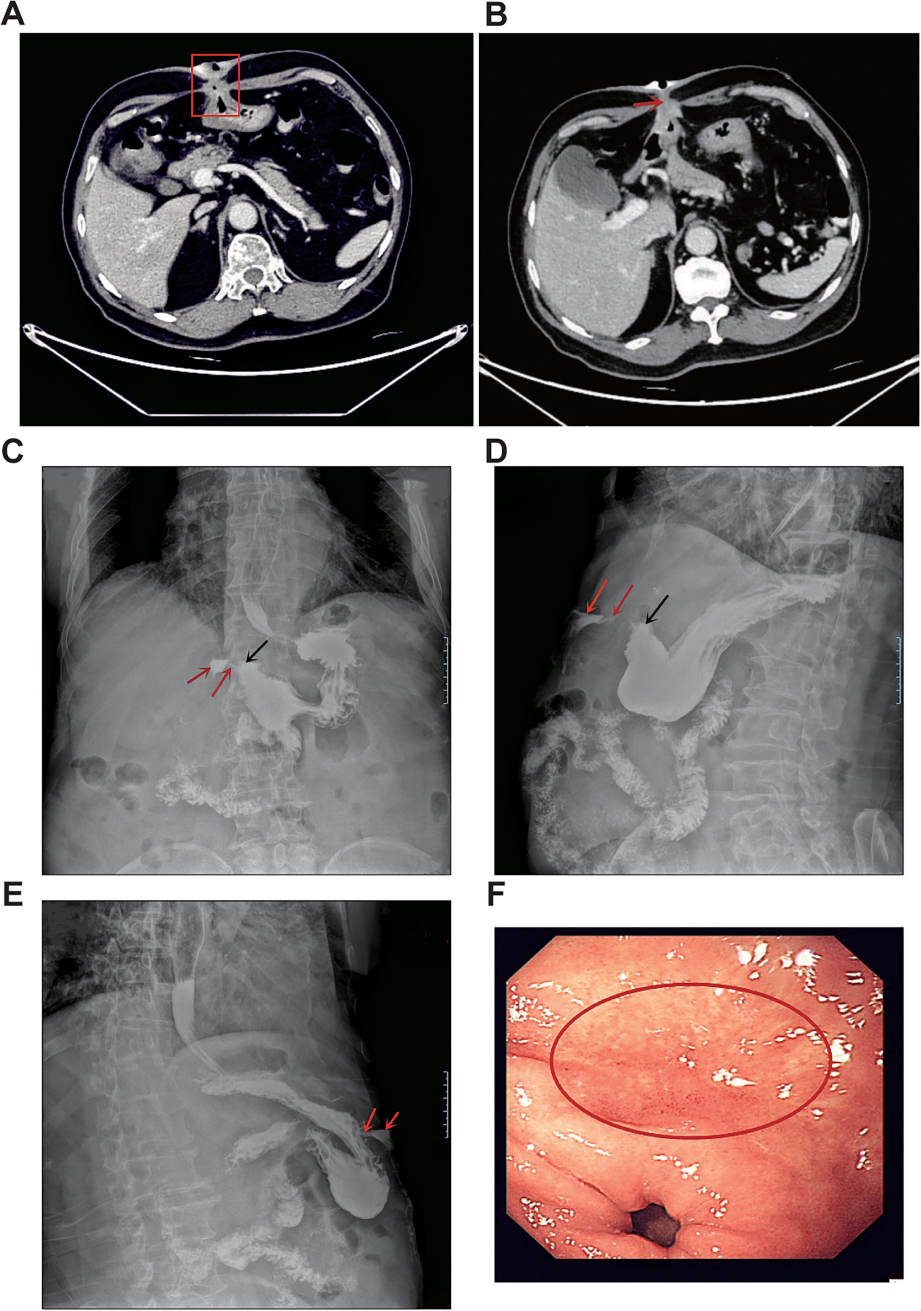
Preoperative imaging and endoscopy. **(A,B)** Contrast-enhanced CT scans. The red box **(A)** and red arrow **(B)** highlight the contiguous gastrocutaneous fistulous tract. **(C–E)** Upper GI contrast study in anteroposterior **(C)**, left lateral **(D)**, and right lateral **(E)** views, showing contrast extravasation from the gastric antrum (black arrows) through the fistula (red arrows). **(F)** Esophagogastroduodenoscopy (EGD) revealing the internal orifice; the red circle indicates an irregular ulcer (1.2 × 0.4 cm) on the antral lesser curvature.

Contrast-enhanced abdominal CT demonstrated obliteration of the normal fat plane between the anterior gastric antrum and the abdominal wall, with clear visualization of a continuous tract connecting the gastric lumen to the skin surface (Figures 2A, B). To further delineate the fistula, an upper gastrointestinal contrast study using a water-soluble agent (Iohexol) was performed. This examination demonstrated normal esophageal passage and a compliant gastric wall with preserved mucosal folds. No additional ulcerations or filling defects were identified. Importantly, contrast extravasation was observed originating from the anterior antral wall and extending through the abdominal wall to the skin, confirming the diagnosis of a gastrocutaneous fistula (Figures 2C–E). Esophagogastroduodenoscopy was subsequently conducted to exclude malignancy. Endoscopic findings included mucosal congestion and bile reflux. A shallow, irregular mucosal depression measuring 1.2 × 0.4 cm was identified along the lesser curvature of the antrum, corresponding to the internal fistula opening (Figure 2F). Multiple targeted biopsies demonstrated chronic inflammatory changes without evidence of dysplasia or malignancy.

### 2.3 Therapeutic intervention

**FIGURE 3:**
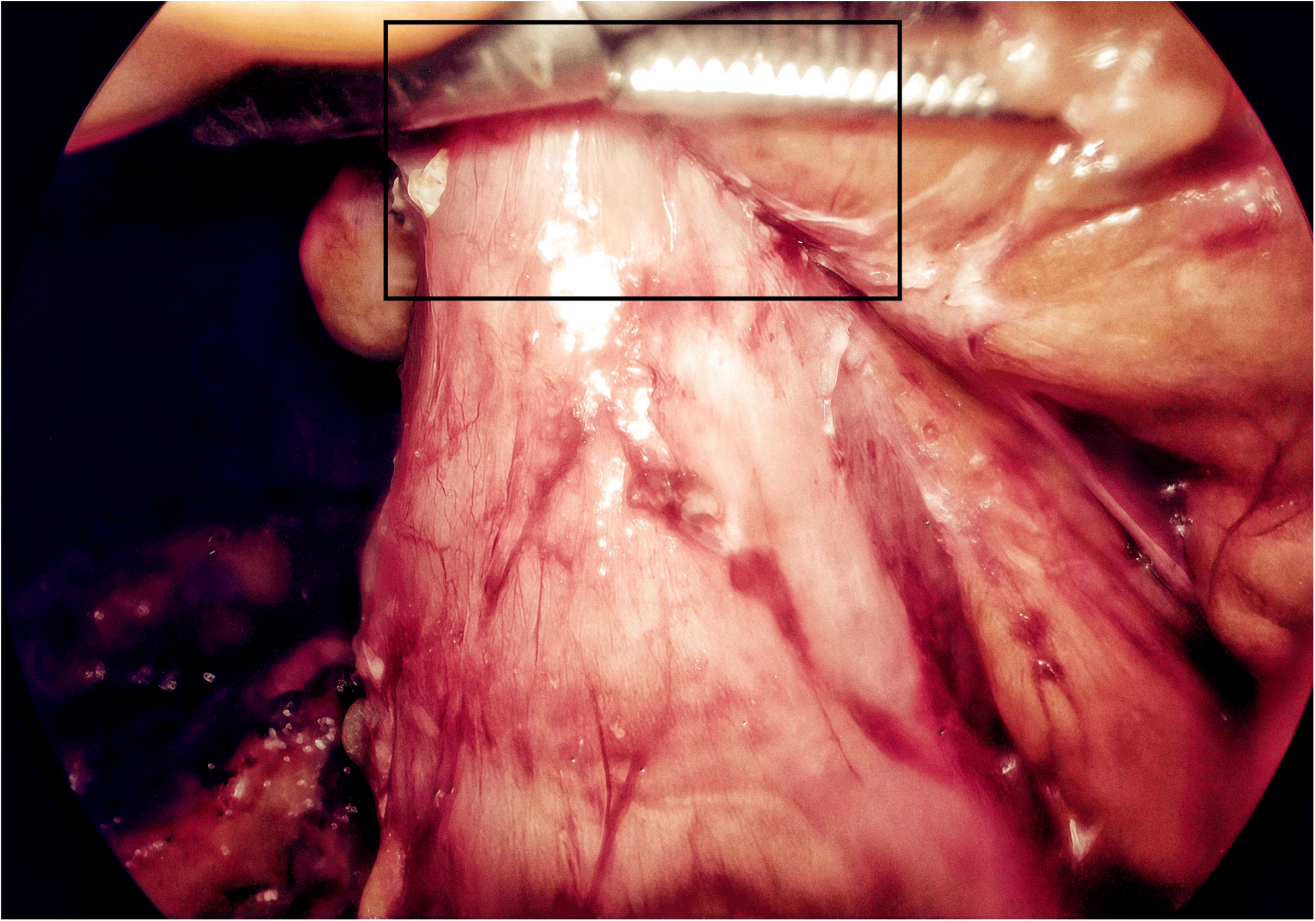
Intraoperative laparoscopic findings. Severe fibroinflammatory adhesions with the greater omentum encapsulating the lesion, forming a dense “walling-off” barricade against the abdominal wall.

After preoperative optimization, including administration of broad-spectrum intravenous antibiotics, bowel rest, and total parenteral nutrition, the patient underwent surgical treatment. Laparoscopic exploration revealed extensive, dense fibroinflammatory adhesions confined to the upper abdomen. The greater omentum was adherent to the lesion, effectively isolating it from the surrounding peritoneal cavity (Figure 3). A firm, indurated lesion on the anterior gastric antrum was found to be tightly adherent to the hepatoduodenal ligament and anterior parietal peritoneum. Given the extent of fibrosis and distortion of local anatomy, primary repair was considered high risk for postoperative leakage. Therefore, a laparoscopically assisted subtotal gastrectomy with Roux-en-Y reconstruction was performed. Simultaneously, a spindle-shaped skin incision approximately 10 cm in length was made to excise the external opening, the entire fistulous tract, and the affected portion of the stomach in a single *en bloc* specimen.

### 2.4 Follow-up and outcomes

**FIGURE 4:**
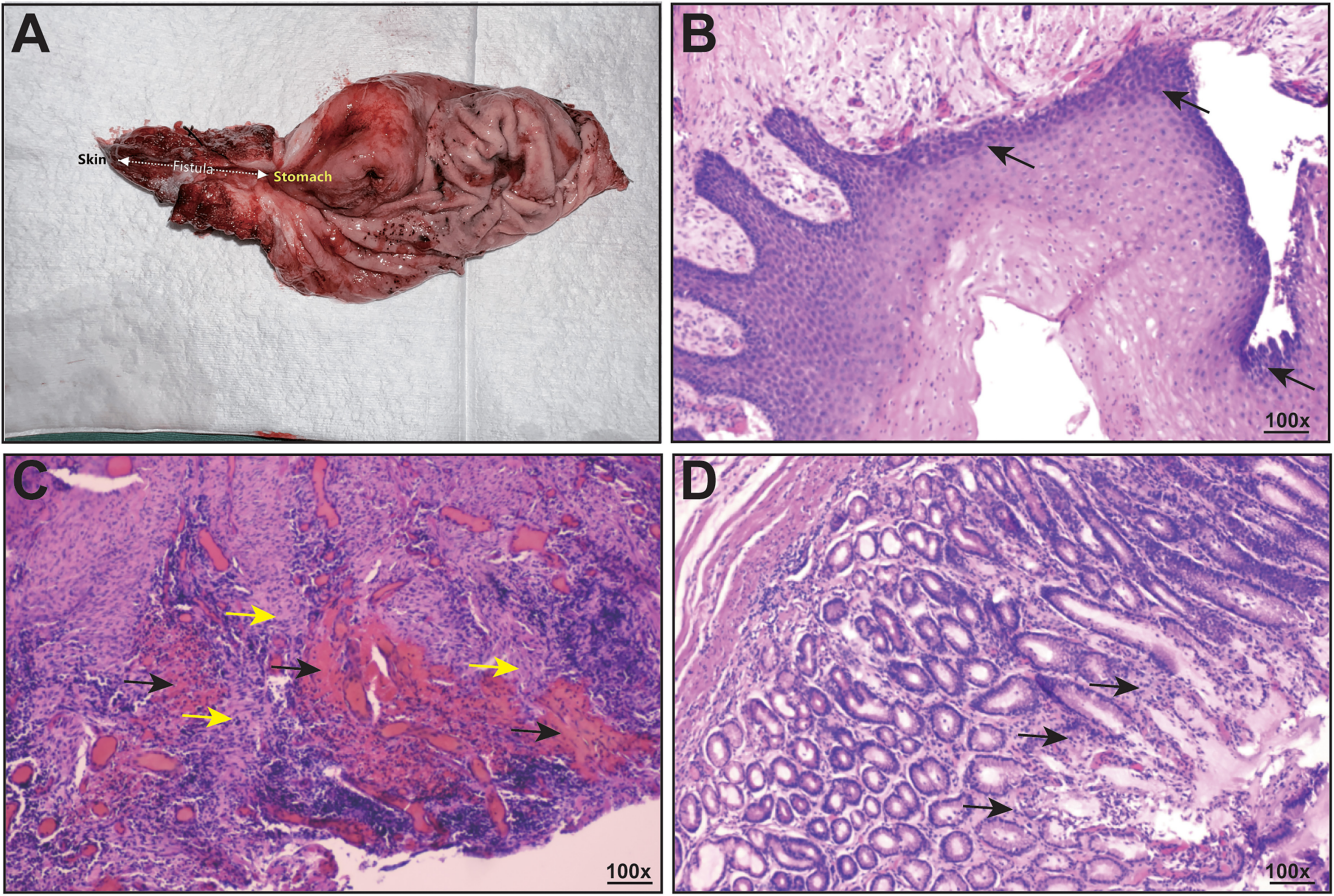
Pathological evaluation of the resected specimen. **(A)** Macroscopic view showing the fistulous tract connecting the gastric mucosa to the abdominal skin. **(B)** Abdominal skin, with the black arrow indicating squamous epithelium. **(C)** Fistula wall exhibiting repair of chronic ulceration, granulation tissue, and hemorrhage (yellow arrow: inflammatory infiltrates; black arrow: neovasculature with hemorrhage). **(D)** Adjacent gastric mucosa showing chronic inflammation and erosion (black arrow: inflammatory infiltrates) (H&E stain).

Gross examination of the resected specimen revealed a well-formed, thick-walled fistulous tract (length, 4.5 cm; width, 2.5 cm), extending from the gastric mucosa to the skin surface (Figure 4A). Histopathological analysis demonstrated a stratified squamous epithelial lining at the external opening (Figure 4B). The wall of the fistulous tract was composed of mature granulation tissue, abundant neovascularization with focal hemorrhage, dense inflammatory infiltrates (Figure 4C), and focal aggregates of histiocytes and foreign body-type multinucleated giant cells. The tract transitioned into a chronic ulcerative lesion at its gastric origin. The adjacent gastric mucosa showed chronic inflammatory and erosive changes (Figure 4D). No evidence of malignancy, tuberculosis, or Crohn’s disease was found, nor were any well-formed granulomas or *H. pylori* organisms identified. Postoperative recovery was uneventful. The surgical wound healed using primary intention, and the patient was discharged on postoperative day 10 with a proton pump inhibitor regimen. At 3-month follow-up, he remained symptom-free with no evidence of recurrence (Table 1).

**TABLE 1:** Timeline of the episode of care.

Date/period	Clinical events and milestones
40 years ago	Open appendectomy for acute appendicitis (McBurney incision).
30 years ago	Patient first noticed a small, painless, stable subcutaneous mass in the epigastric region.
1 month ago	Accidental trauma to the nodule; local skin breakdown and persistent enteric-like fluid discharge (500 mL/day).
Day 1 (admission)	Physical examination and laboratory tests; fluid collection started.
Day 2–3	Contrast-enhanced CT, upper GI contrast study (Iohexol), and esophagogastroduodenoscopy (EGD) with biopsy.
Day 5	Preoperative optimization (bowel rest, TPN, intravenous antibiotics).
Day 7	Laparoscopic-assisted subtotal gastrectomy with Roux-en-Y reconstruction and *en bloc* excision of the fistula.
Day 17 (post-op day 10)	Uneventful recovery; surgical wound healed; discharged with PPI regimen.
3 months later	Follow-up visit: symptom-free, no evidence of recurrence.

## 3 Discussion

### 3.1 Etiology and pathogenesis

Available evidence indicates that benign spontaneous gastrocutaneous fistulas are extraordinarily uncommon. A systematic review of gastrointestinal fistulas reported that spontaneous cases unrelated to either medical intervention or malignancy account for fewer than 1% of all documented instances (9).

The mechanism underlying the presentation in this patient is particularly noteworthy and raises an important physiological question: why did the disease not culminate in an acute free perforation with diffuse peritonitis? As noted by Søreide et al., the precise biological determinants that dictate whether an ulcer will undergo free perforation, remain quiescent, or penetrate adjacent structures have not yet been fully elucidated (2). Despite this biological uncertainty, relevant studies, such as that by Pickhardt et al., have emphasized that the development of such a fistula is associated with a sustained inflammatory response, resulting in the establishment of dense serosal adhesions that create a biological barrier before full-thickness mural penetration (7). In chronic inflammatory disease, a GCF can be precipitated by inflammatory erosion of the gastric wall, leading to the development of an abscess and ultimately formation of a fistula (10). This process depends on two essential conditions: (1) a penetrating anterior gastric ulcer, and (2) pre-existing or subsequently formed adhesions between the stomach and the anterior abdominal wall. It should be noted that due to the extreme rarity of this condition in the era of modern anti-ulcer therapy, the current pathophysiological understanding remains primarily rooted in these foundational evidenced-based studies.

A comparative analysis of existing literature on SGCF attributable to benign gastric ulceration provides a stronger foundation for this mechanistic sequence (11–13), suggesting that fistulization can result from chronic inflammatory changes rather than malignancy. Although the acute spontaneous case described by Saïdou et al. (12) and our 30-year indolent case exhibit fundamentally different natural histories, both share the same fistulizing endpoint. This common outcome indicates that avoidance of free peritonitis depends primarily on immediate localized anatomical containment (i.e., rapid serosal adhesion) at the exact time of penetration onset, rather than solely on a prolonged timeline. In acute scenarios, rapid fibrinous exudation induces fragile adhesions that can temporarily prevent diffuse spillage. In contrast, the extended timeline of the present patient enabled repeated micro-penetrations following this initial containment, ultimately resulting in the formation of a thick, fibrotic, and epithelialized tract. Notably, there is no definitive explanation in the literature on why certain ulcers are successful in triggering this protective localized response while others progress rapidly to peritonitis. This represents an unresolved gap in current surgical knowledge.

Although the patient had undergone an appendectomy four decades earlier, the remoteness of the appendectomy incision suggests that it was not a likely explanation of the highly localized upper abdominal containment observed in this case. Instead, the prolonged and indolent course more plausibly promoted repeated low-grade peritoneal inflammation, progressive adherence of the greater omentum and parietal peritoneum to the diseased gastric segment (14), and eventual formation of a mature fistulous tract. The recent mechanical injury to the overlying skin likely disrupted the final barrier, converting a previously contained sinus into a fully developed gastrocutaneous fistula.

### 3.2 Diagnostic workup

From a diagnostic standpoint, the foremost priority in evaluating a spontaneous abdominal wall fistula is the definitive exclusion of malignancy. Advanced gastric cancers, gastrointestinal stromal tumors, and lymphomas represent the most frequent intrinsic causes of such fistulas, as tumor necrosis can lead to tract formation (7). In addition, granulomatous conditions, including Crohn’s disease (15), tuberculosis (16), and actinomycosis, must be carefully considered during differential diagnosis.

Cross-sectional imaging, particularly contrast-enhanced CT with fistulography, plays a central role by clearly delineating the origin, trajectory, and anatomical relationships of the fistulous tract (10). However, imaging alone is insufficient to reliably distinguish benign from malignant causes. Therefore, endoscopic assessment with targeted, deep biopsies from the internal opening is indispensable for establishing a definitive preoperative diagnosis. In this case, the integration of imaging and endoscopic findings provided a clear and reliable diagnostic pathway.

### 3.3 Therapeutic strategies

A systematic comparison of the management strategies described in existing case reports revealed that the therapeutic choices for SGCF are determined by the chronicity, underlying etiology, and size of the fistula.

Management of gastrocutaneous fistulas generally begins with conservative measures (17), including bowel rest, total parenteral nutrition, proton pump inhibitor therapy, and the use of somatostatin analogues such as octreotide to suppress gastrointestinal secretions (18). This approach is typically reserved for acute, small, non-epithelialized fistulas where spontaneous closure remains a possibility. More recently, minimally invasive endoscopic techniques, such as over-the-scope clips and fibrin sealants, have been introduced as alternative treatment options (19, 20). However, these approaches are primarily effective in acute, small (<1–1.5 cm), and non-epithelialized fistulas, particularly those of iatrogenic origin, such as those that develop after PEG tube removal. Surgical management represents the definitive therapeutic approach for mature, fibrotic, or medically refractory fistulas (11), while simple fistulectomy with primary gastric repair was described in cases with limited inflammation.

In the present case, both conservative and endoscopic strategies were unlikely to succeed for several reasons. First, the defect size (2.5 cm in diameter) exceeded the closure capacity of currently available endoscopic devices. Second, the underlying pathology involved a substantial chronic gastric ulcer (approximately 4 cm in diameter), which required surgical removal to definitively exclude malignancy. Third, the long-standing nature of the lesion resulted in a rigid, fibrotic tract that is inherently resistant to non-surgical closure (10, 21), and primary suture repair of such indurated tissue would carry an unacceptable risk of dehiscence.

Histological examination confirmed a mature, thick-walled tract lined with granulation tissue and characterized by a multinucleated giant cell response. Existing evidence indicates that such well-established, fibrotic fistulas are unlikely to close spontaneously due to persistent exposure to gastric secretions and the structural rigidity of the tract (3, 22, 23). While some reports describe success with simple fistulectomy and primary gastric repair, this tissue-sparing approach carries a significant risk of recurrence and leakage in the setting of extensive fibrosis and chronic inflammation. Accordingly, a more reliable strategy involves en bloc resection of the entire fistulous complex, including the cutaneous opening, the tract, and the involved gastric segment. In the present patient, a subtotal gastrectomy with tension-free Roux-en-Y reconstruction provided a definitive and durable solution, distinguishing this intervention from simpler strategies used for more acute or less extensive lesions.

This unique presentation also raises a critical clinical question regarding the management of similar lesions if they are diagnosed before cutaneous rupture. Even in asymptomatic cases, conservative observation is associated with substantial hazards, including the risk of future traumatic fistulization, intra-abdominal infection, and missed malignancy, particularly as superficial endoscopic biopsies may not rule out deep-seated neoplastic processes. Therefore, we recommend proactive surgical resection once a fistulous connection is suspected. Elective *en bloc* resection not only preempts unpredictable clinical complications but also ensures the acquisition of a full-thickness specimen for definitive histopathological confirmation.

### 3.4 Strengths and limitations

A key strength of this report is the thorough diagnostic evaluation, incorporating contrast imaging, three-dimensional CT reconstruction, endoscopy with biopsy, and definitive histopathological analysis, all of which collectively confirm this rare diagnosis. Additionally, the unusually long 30-year clinical course, when compared with the few reported cases of chronic gastric ulcer-related SGCF, provides a rare clinical illustration of a prolonged, localized fistulizing course. However, this study is inherently limited by its single-case design. Broader conclusions regarding optimal management and long-term outcomes will require accumulation and analysis of additional cases with similar presentations.

## 4 Conclusion

Spontaneous gastrocutaneous fistula originating from a benign gastric ulcer is an exceptionally rare clinical entity that can present as an indolent, long-standing abdominal wall lesion. The imaging, operative, and histopathological findings in the present case support the interpretation that chronic localized inflammation and adhesion formation may facilitate cutaneous fistulization while limiting free intraperitoneal perforation. Gastrointestinal fistulas should be suspected when chronic abdominal wall lesions develop enteric-like drainage. After careful exclusion of malignancy, mature fibrotic fistulas of this type generally require definitive *en bloc* surgical resection for durable resolution.

## Data Availability

The original contributions presented in this study are included in this article/supplementary material, further inquiries can be directed to the corresponding author.
